# Modeling Melanoma *In Vitro* and *In Vivo*

**DOI:** 10.3390/healthcare2010027

**Published:** 2013-12-23

**Authors:** Kimberley A. Beaumont, Nethia Mohana-Kumaran, Nikolas K. Haass

**Affiliations:** 1 The Centenary Institute, Newtown, New South Wales 2042, Australia; E-Mails: k.beaumont@centenary.org.au (K.A.B.); nethiakumaran@usm.my (N.M.-K.); 2 School of Biological Sciences, Universiti Sains Malaysia, 11800 Georgetown, Penang, Malaysia; 3 Discipline of Dermatology, University of Sydney, New South Wales 2006, Australia; 4 The University of Queensland Diamantina Institute, Translational Research Institute, The University of Queensland, Brisbane, Queensland 4102, Australia

**Keywords:** melanoma, 3D models, spheroid models, animal models, xenograft models, genetically engineered mouse models (GEM), zebrafish models

## Abstract

The behavior of melanoma cells has traditionally been studied *in vitro* in two-dimensional cell culture with cells adhering to plastic dishes. However, in order to mimic the three-dimensional architecture of a melanoma, as well as its interactions with the tumor microenvironment, there has been the need for more physiologically relevant models. This has been achieved by designing 3D *in vitro* models of melanoma, such as melanoma spheroids embedded in extracellular matrix or organotypic skin reconstructs. *In vivo* melanoma models have typically relied on the growth of tumor xenografts in immunocompromised mice. Several genetically engineered mouse models have now been developed which allow the generation of spontaneous melanoma. Melanoma models have also been established in other species such as zebrafish, which are more conducive to imaging and high throughput studies. We will discuss these models as well as novel techniques that are relevant to the study of the molecular mechanisms underlying melanoma progression.

## 1. Introduction

Melanoma is the most aggressive and deadly form of skin cancer. Patients with distant metastases have a five-year survival rate of 16% [1] and a median survival of four to six months [2]. Until very recently, melanoma has been branded by the failure of chemotherapy and other therapeutic attempts. However, the discovery that 40%–50% of melanomas harbor activating BRAF mutations [3,4,5] prompted the development of selective BRAF inhibitors. The first specific ones were the lead compound PLX4720 [6], and the pharmacokinetically superior PLX4032/vemurafenib [7]. The extraordinarily quick bench-to-bedside history of this drug is a great example of using the appropriate melanoma models at different steps of preclinical development. Targeting oncogenic BRAF with PLX4720 or PLX4032 resulted in inhibition of growth and invasion of three-dimensional melanoma spheroids into a collagen matrix and caused tumor regression of melanoma xenografts without evidence of toxicity [6,7]. This was mirrored in phase II and phase III patient trials and has finally lead FDA-approval of vemurafenib; however, despite these unprecedented response rates, rapid onset of resistance is a major issue [4,5,8].

The interaction of cancer cells with the tumor microenvironment plays a major role in the function and regulation of cancer cells and is therefore a critical determinant of the response of cancer cells to therapeutic agents [9,10]. An important feature of a melanoma model system is that melanoma cells recapitulate their proliferative, migratory, and invasive properties. The ability to maintain features of the primary tumor can as well as tumor progression aid in the selection of the model system in which to study melanoma cells. The goal is to recreate, as closely as possible, the features of the tumor in the proper microenvironmental context. However, it is also important to consider factors, such as ease of use, data interpretation, cost, and applicability issues, such as genetic manipulation and drug delivery, when selecting a model system [11]. Here we will discuss the major model systems and their application in the study of melanoma.

## 2. Two-Dimensional Cell Culture

**Figure 1:**
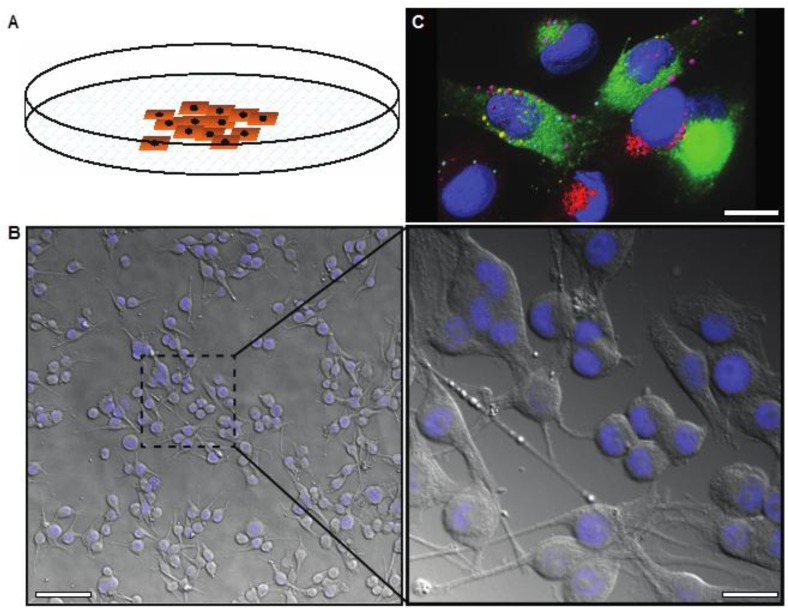
(**A**) Image showing two-dimensional cell growth in adherent cell culture in a plastic culture dish; (**B**) Differential interference contrast image overlaid with DAPI stain (nuclei, blue) demonstrating the morphology of melanoma cells in 2D culture, low and high magnification; (**C**) Deconvolved immunofluorescence image representing the highly complex internal structure of melanoma cells, labeled with markers for different endosomes or secretory vesicles (pink, red, or green) and the nucleus (blue). Scale bars: **(B**) 50 μm (left), 12 μm (right); (**C**) 6 μm.

Most studies of melanoma cell biology and anti-melanoma drug activity have come from work done with two-dimensional (2D) adherent cell culture assays. Under these conditions, the cells are usually grown as monocultures on plastic tissue culture plates (Figure 1A,B). The principal advantages of such models are simplicity, convenience, and cost. The cells are grown in the manner to which they have been accustomed with maximal access to nutrients, oxygen, and applied drugs. It is often the case that drugs that do not work in 2D culture have no effect in more realistic models, thus, 2D assays are useful and required for preliminary high-throughput screens before employing more sophisticated preclinical models. There are of course drugs that rely on poor access to nutrients and oxygen, or upon metabolic conversion in an animal (such as dacarbazine), or which stimulate the immune response (e.g., interferons). 2D models are of no help in these cases. 

In general, conventional 2D culture is utilized for selected assays, such as protein-expression, cytotoxicity, migration and adhesion assays. For example, the effect of drugs on proliferation is monitored by manual or automatic cell counting or by high throughput colorimetric or fluorescence assays based on metabolic activity or DNA content. Cell migration and invasion are assayed by the simple scratch (wound closure) and Boyden chamber assays, respectively. To study effects on different adhesion molecules [12,13], culture plates can be coated with the appropriate matrices.

Cells in 2D culture are easily visualized using conventional light microscopy (Figure 1B). There are no problems with light penetration, which can occur in thicker samples, and no autofluorescence due to other non-cellular structures that occur in tissue samples. Live imaging is also relatively simple with the use of humidified, temperature and gas controlled imaging chambers. Further, growing cells on thin glass coverslips or imaging plates allows optical sectioning and 3D rendering of fluorescently tagged cells/proteins can be performed using confocal or wide field deconvolution microscopy (Figure 1C). Intracellular trafficking, in particular aberrant protein trafficking, is becoming increasingly implicated in cancer cell biology, including melanoma [14,15]. High-resolution, live cell imaging is necessary in order to analyze these dynamic processes, as well as other dynamic processes important in cancer progression such as cell migration and invasion.

Monoculture experiments have the enormous advantage that they are pure and free from contaminating cells important for protein, RNA or DNA extraction. However, other cells in the microenvironment can influence the RNA expression or protein synthesis patterns. To allow for that, co-culture experiments are of advantage—this is especially important when studying the biology of melanocytes. While melanocytes in monoculture show aberrant proliferation behavior, morphology and RNA expression, or protein synthesis patterns, in co-culture with keratinocytes they exhibit a phenotype much more similar to that *in vivo* [16,17].

## 3. Three-Dimensional Cell Culture

Although adherent cell culture models are useful for investigating basic principles of tumor cell biology, they do not take into consideration that melanoma cells do not grow in isolation. Instead they are oriented in a three-dimensional space, establishing continuous dynamic interaction with the stroma, *i.e.*, extracellular matrix and other cell types such as endothelial cells, fibroblasts, and immune cells. This is often referred to as the ‘tumor organ’ [18,19]. Therefore, growing melanoma cells in culture flasks is not always an adequate and reliable system to study melanoma biology and drug resistance [20,21,22]. Three-dimensional (3D) cultures represent a good compromise between the lack of a microenvironment encountered under 2D culture conditions and the great complexity of the *in vivo* animal models [18]. 3D cultures are inexpensive and less time-consuming than animal studies and are therefore a useful tool to prescreen single agent drugs and drug combinations. 3D models allow the researcher to narrow down the experiments that need to be done in animals and thus to reduce the number of animals used in preclinical studies. This has both an ethical and economical advantage. 

### 3.1. 3D Spheroid Model

**Figure 2:**
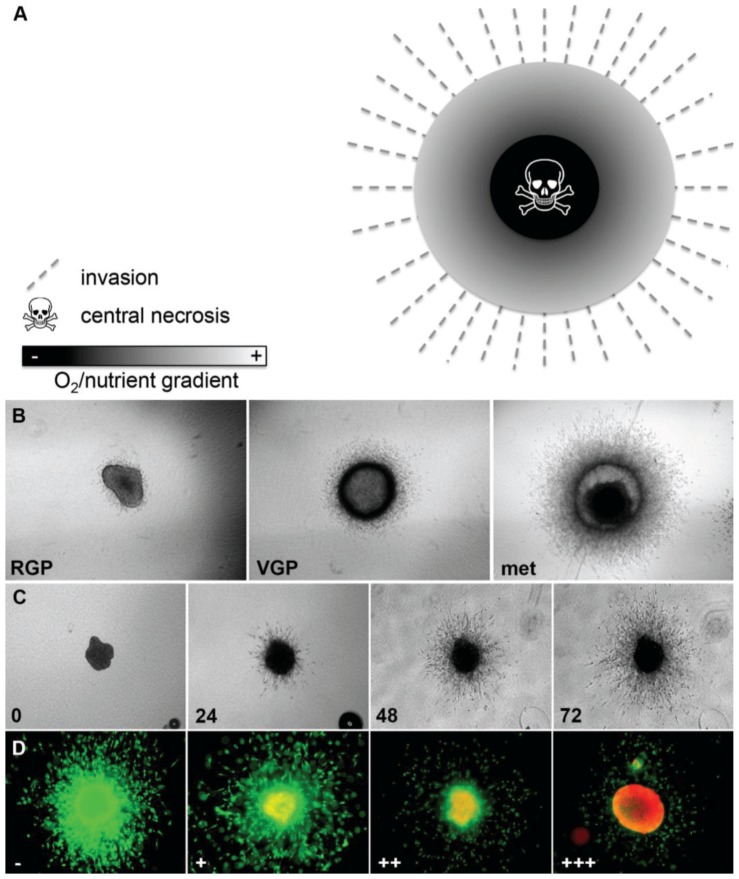
Melanoma spheroid model. (**A**) Image demonstrating oxygen and nutrient gradient within a 3D spheroid, central necrosis and invasion of melanoma cells into the tumor stroma; (**B**) Growth and invasion behavior of melanoma spheroids reflects that of the original tumors: radial (RGP), vertical growth phase (VGP), metastasis (met); (**C**) Growth and invasion of a spheroid derived from a metastatic cell line (numbers: time in h); (**D**) Spheroid treated with increasing doses of the BRAF inhibitor vemurafenib (−, +, ++, +++) and stained with a live/dead assay (green: calcein-AM, live; red: ethidium bromide, dead). Note the increasing growth and invasion inhibition, as well as cell death.

3D melanoma spheroids implanted into a collagen gel matrix mirror the *in vivo* tumor architecture and microenvironment more closely than adherent cell culture [11,18,23]. We have previously described the generation of spheroids using the liquid overlay method and the technical aspects of this model in detail [18,23]. This model mimics the tumor heterogeneity seen *in vivo* as it recreates the oxygen/nutrient gradient with a hypoxic zone and a central necrosis and allows interaction between melanoma cells and their stroma (Figure 2A). For example, activity of the extracellular signal-regulated kinase (ERK), which indicates proliferative activity, is homogeneous in 2D culture and can be inhibited totally through treatment with small-molecule inhibitors of the MAPK pathway [24]. In contrast, in 3D spheroids ERK-activity is mainly found in the growing periphery [24], similar to melanoma lesions in patients [25], indicating that this heterogeneous sub-compartmental expression of active signaling molecules in solid tumors will have important implications for the successful translation of novel targeted therapies or combinations. Furthermore, the 3D spheroid model faithfully recapitulates the behavior of melanomas *in vivo* in that cell lines of different origin (*i.e.*, primary tumors, radial (RGP) and vertical growth phase (VGP), and metastasis-derived cells) display different growth and invasion characteristics reflecting the original state of aggressiveness (Figure 2B) [23]. This close resemblance to the situation encountered *in vivo* facilitates more realistic study of melanoma growth, invasion (Figure 2C), and drug response (Figure 2D and Figure 5A,B). For example, targeting oncogenic BRAF with PLX4720 or PLX4032 resulted in inhibition of growth and invasion of 3D spheroids and caused tumor regression of melanoma xenografts [6,7], which was mirrored in phase II and phase III patient trials and has finally lead FDA-approval of vemurafenib [4,26]. Similarly, we have utilized the spheroid model to study the contribution of zonula occludens protein 1 (ZO-1) to the oncogenic behavior of melanoma [27], the response to MEK-inhibitors (MAPK pathway) and/or PI3K-inhibitors (PI3K/AKT/mTOR pathway) [23,24], BH3-mimetics (intrinsic apoptosis pathway) [20,21], and a novel class of anti-tropomyosin compounds (cytoskeleton) [28]. While in most of these studies 72-h spheroid assays predicted the outcome in xenograft studies *in vivo* [6,7,24,28], this did not seem to be the case in the BH3-mimetic study [20]. To model the growth of xenografts that did *not* respond to the drug, spheroids were here allowed seven days growth before treatment with the BH3-mimetic ABT-737, resulting in a noticeable effect on viability in the *periphery* of the spheroids but *not the center*, suggesting that diffusion of this particular drug into larger melanoma masses may be limiting [20]. Thus, this model can be utilized to study both drug efficacy and bioavailability.

Another advantage of the 3D spheroid model is its versatility. The collagen gel itself can be easily manipulated to alter elasticity and stiffness. Increased substrate stiffness results in activation of the non-receptor focal adhesion kinase (FAK) and small GTP binding protein (RhoGTPase) pathways, leading to increased cell proliferation and invasive phenotype changes including changes in gene expression [29].

There are alternative methods. For example, Kramer and colleagues described a modified spheroid migration assay, in which tumor spheroids are formed and applied to confluent fibroblasts (e.g., cancer associated fibroblasts, CAFs), which are grown on a conventional plastic dish. This ‘2D/3D hybrid’ model can be used to analyze the migratory changes upon interaction between the two cell types [30]. Ghajar and colleagues have used fibrin hydrogels instead of collagen I gels for 3D spheroid studies [31]. The basement membrane matrix secreted by Engelbreth-Holm-Swarm (EHS) mouse sarcoma cells (Matrigel^®^, San Jose, CA, USA) is commonly used for 3D culture assays. Its heterogeneous composition is an advantage and disadvantage at the same time. Advantage because it provides all components to create an ideal environment for cancer cells. Disadvantage because this heterogeneity is difficult to control. In contrast, the above-described collagen I system is well defined. If additional components (e.g., certain growth factors or even other additional cell types) are needed, they can be easily added to the system [32].

### 3.2. Tumor Sphere Model

Growth of melanoma spheres was initially developed because it was thought that the stem cell conditions would enrich an otherwise small ‘cancer stem cell’ population [33,34]. Whether these conditions are really enriching for true melanoma cancer stem cells is controversial [35]. However, growing melanoma cells as spheres has been shown to increase tumorigenicity and heterogeneity. Long-term non-adherent growth of cells in “spheres” is achieved via growth of cells in non-adherent plates [36], or via spontaneous formation of spheres in adherent monoculture in the presence of stem cell media [33,37]. Similar to the spheroid model—melanoma spheres can also be implanted into different matrices to assay melanoma growth and invasion.

### 3.3. 3D Skin Reconstruct Model

**Figure 3:**
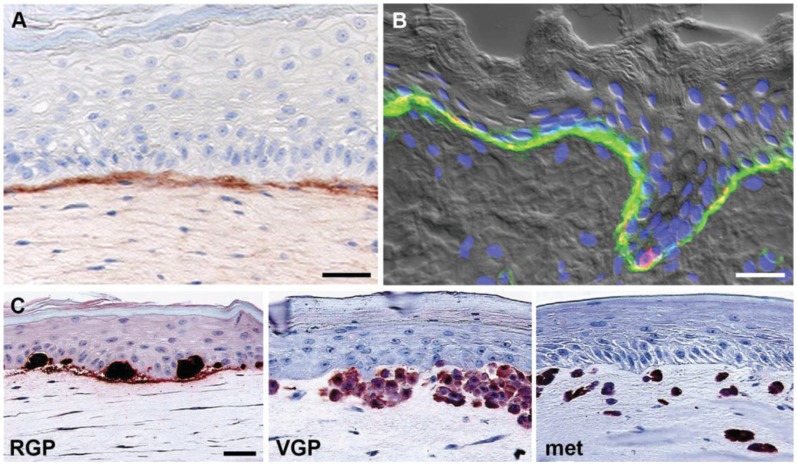
3D skin reconstruct model. (**A**) Model: Keratinocytes and fibroblasts in human skin reconstructs produce a basement membrane (stained with a monoclonal antibody against collagen IV). Note the clear demarcation of the basement membrane; (**B**) Skin: Human fetal foreskin stained with a monoclonal antibody against α6 integrin (green) and the melanocyte marker HMB45 (red), highlighting the continuous basement membrane between epidermis including basal keratinocytes and melanocytes (co-localization, yellow) and dermis. Note the similarity between model and original; (**C**) Melanoma cells recapitulate original tumor phenotype in reconstructed skin. Melanoma cells were stained for S100. Radial growth phase (RGP) melanoma cells proliferate and form nests in the epidermis, but do not invade the dermis. Vertical growth phase (VGP) cells cross the basement membrane, invade and proliferate in the dermis. Cells from metastatic (met) melanoma rapidly invade deep into the dermal compartment. Figure adapted from Haass *et al*., 2005 [12] and Santiago-Walker *et al.*, 2009 [11]. Scale bars: 30 μm.

Skin reconstructs consist of artificial skin rebuilt from isolated cell populations and composed of a stratified, terminally differentiated epidermal compartment of keratinocytes and melanocytes, a dermal compartment consisting of fibroblasts embedded in collagen, and a well established basement membrane deposited by skin cells (Figure 3A) [38,39]. We have previously described the generation of 3D skin reconstructs and the technical aspects of this model in detail [18]. Reconstructed skin closely resembles histologically human skin (Figure 3B) in architecture and composition, with all major cell types represented in physiologically relevant ratios. In human skin reconstructs, melanoma cells from different stages of progression have the same properties as in the patients’ skin, *i.e.*, cells derived from a melanoma *in situ*/RGP are unable to invade the dermis from the epidermis, whereas advanced primary (VGP) and metastatic melanoma cells readily invade the dermis (Figure 3C) [12,38,39]. A number of recent studies have assessed the effects of novel anti-cancer drugs in 3D skin reconstructs. For example, we made use of this model to show that selective BRAF-inhibitors (PLX4720 and vemurafenib) are capable of decreasing proliferation, as well as inducing apoptosis in mutant BRAF melanomas, while non-transformed cells were unaffected indicating that therapeutic toxicities in patients may be minimal [6,7]. Meier and colleagues reported that combinations of MAPK and AKT inhibitors completely suppressed invasive tumor growth of melanoma cells in a similar model of regenerated human skin [40].

These 3D skin reconstruct models, which are technically demanding and require constant monitoring by trained personnel during the prolonged culture times of up to 21 days, are mainly used for studying the biology and drug response of melanoma cells at early stages, *i.e.*, when they are still part of the cross-talk between epidermis and dermis. In contrast, the above-discussed 3D spheroid model is used for high-throughput proliferation, invasion and drug response assays mimicking distant metastatic melanoma, where the context to the skin is lost.

### 3.4. 3D Neoangiogenesis Model

**Figure 4:**
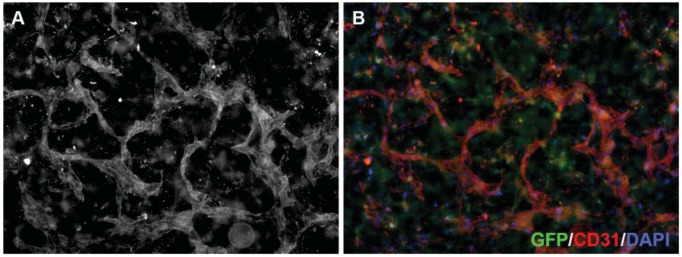
3D neoangiogenesis model. Human microvascular endothelial cells grown in a collagen gel containing green fluorescent protein (GFP)-expressing fibroblasts (green in **B**) for 144 h to allow the formation of a three-dimensional vascular network, were fixed and incubated with an antibody against CD31 (**A**; red in **B**) and stained with DAPI (blue in **B**). Tube formation and branching frequency are used to quantify anti-angiogenic drug effects.

Oxygen and nutrient supply is an important factor for solid tumor cell survival and proliferation [41,42]. Tumors quickly grow beyond the reach of the physiological blood supply and thus form their own tumor vasculature. This complex process, driven by the secretion of pro-angiogenic factors from the tumor cells themselves and from the tumor stroma cells, is called neoangiogenesis. These tumor blood vessels are—in contrast to normal blood vessels—disorganized, tortuous and often leaky [43]. Many cancer therapies are directed against the tumor vasculature. The common view is that anti-angiogenic therapy should destroy the tumor vasculature, thereby depriving the tumor of oxygen and nutrients. However, there is emerging evidence supporting the hypothesis that anti-angiogenic agents can also transiently ‘normalize’ the abnormal structure and function of tumor vasculature to make it more efficient for oxygen and drug delivery [43]. It is, thus, very important to assess on- or also off-target anti-angiogenic activity of novel drugs. This can be addressed *in vitro* using the 3D neoangiogenesis model, the generation and technical aspects of which have previously been described in detail [18,44]. As early MAPK pathway inhibitors were known to be multikinase inhibitors that also affected (neo)angiogenesis (e.g., sorafenib inhibits not only RAF but also VEGFR (Vascular Endothelial Growth Factor Receptor) and PDGFR (Platelet Derived Growth Factor Receptor)), we utilized this model to show that the MEK inhibitor AZD6244 despite strong activity in melanoma cells has no significant effect on angiogenesis (Figure 4) [24].

## 4. Xenograft Models

**Figure 5:**
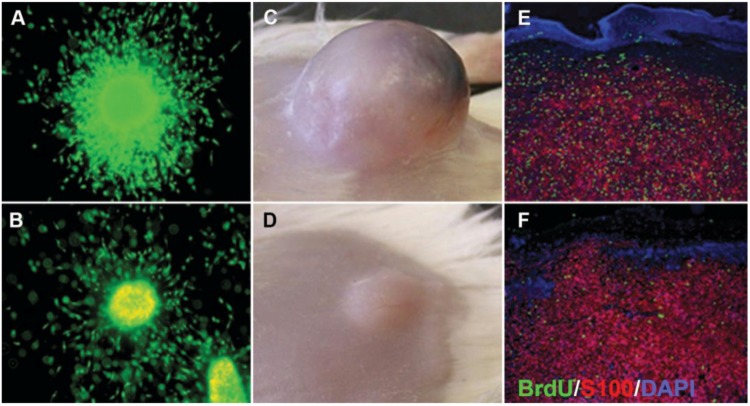
Comparison of 3D melanoma spheroid and xenograft. (**A**) Untreated spheroid; (**B**) spheroid treated with 10 μM selumetinib (MEK1/2 inhibitor AZD6244; Astra Zeneca) and stained with a live/dead assay (green: calcein-AM, live; red: ethidium bromide, dead). Note growth and invasion inhibition as well as increased cell death in the treated spheroid; (**C**) Untreated xenograft; (**D**) xenograft after treatment with 50 mg/kg selumetinib. Note the growth inhibition of the treated tumor. Sections of an untreated (**E**) and treated (**F**) xenograft stained for BrdU (green; proliferation marker), S100 (red, used here as a melanoma marker), and DAPI (blue, nuclear marker) showing a decrease in BrdU uptake after selumetinib treatment. Figure adapted from Haass *et al*., 2008 [24].

Preclinical testing of anti-cancer drugs currently employs an approach in which efficacy endpoints are determined by the growth responses of established human melanoma cell lines after subcutaneous engraftment into immunocompromised mice (cell line xenografts) [45]. This model allows human melanoma cells to directly establish interactions with the murine stroma, including lymphatic and blood vessels and therefore allows the investigation of growth behavior and drug response of human melanoma cells *in vivo* (Figure 5). Moreover, utilization of standardized techniques (*i.e*., same number, passage and culture conditions of injected cells) allows relative control over the timing of tumor growth and the time points of drug administration leading to easily comparable data. Most commonly cells are injected subcutaneously and less frequently intradermally. While the latter reflects the situation of a primary melanoma it has the disadvantage that tumor formation can quickly cause ulceration of the thin mouse skin and therefore to forced termination of the experiment. Subcutaneous injection leads to a tumor more comparable to a skin metastasis. 

Some cell lines metastasize spontaneously from these primary xenograft sites to distant sites such as the lung, so that this model can also be used to study spontaneous metastasis. However, not all cells of a subcutaneous xenograft have the capacity to metastasize readily. Isolation of cells that have spontaneously metastasized to the lung and re-grafted subcutaneously for several passages through immunosuppressed mice leads to selection for an aggressive metastatic sub-population of the originating cell line (e.g., 1,205 Lu and 451 Lu are selected aggressive metastatic sub-lines of WM793B and WM164, respectively). These studies demonstrate that melanoma cells, derived from a heterogeneous population, have distinct phenotypic characteristics, suggesting that metastases are produced by the selective growth of specialized aggressive subpopulations of metastatic cells that pre-existed in the parent tumor [46,47].

Alternatively, tumor cells can be injected into the tail vein to force ‘metastasis’ to the lungs. However, although this approach is frequently used as a metastasis model, it does not represent the actual events of metastasis in a patient scenario, as the first steps of metastasis from the primary tumor into lymph or blood vessels are bypassed in this model [48].

The limitation of established cell lines is that due to continuous selection they eventually differ significantly from the originating cells. As a consequence cell line xenograft models are often poorly predictive of clinical outcome, and drugs tested positive in this model often fail in clinical trials [45]. Hence, more recently it has become a standard to use primary melanoma cells rather than established melanoma cell lines for xenografting. To determine the percentage of tumor-initiating melanoma cells, numbers of primary melanoma cells have been titered down to single cells to generate xenografts in NOD/SCID IL-2 receptor gamma chain knockout (NSG) mice [49]. Another technique is the orthotopic patient-derived xenograft, in which fresh biopsy samples are implanted subcutaneously into NSG mice. By creating avatars of a patient’s melanoma that has relapsed on a drug, ‘mini human-in-mouse trials’ or ‘co-clinical trials’ can be conducted to facilitate the selection of effective drugs, drug combinations, and dosing regimens for that specific patient [45].

Melanoma xenografts, whether they use established cell lines or primary tumor cells, subject transplanted cells to strong selection for defective apoptosis. Moreover, xenografts do not grow in their natural tissue setting. Even orthotopic xenograft models are approximations that do not perfectly reproduce the microenvironment of a tumor during its development. Similarly, xenografts require immunodeficient hosts and, thus, do not grow in the context of an intact immune system. This can be overcome by utilizing a syngeneic xenograft model. The most widely used syngeneic model is the B16 cell line series, which was originally derived from a chemically induced melanoma arising in C57BL/6J mice [50]. While this model is good for studying immune response, its major disadvantage is the use of murine melanoma cell lines, which are limited in their range and are not a good match for the human counterparts. The B16 model is limited due to it being derived from an inbred mouse strain with little genetic diversity. In addition, mouse melanoma cells differ from human melanoma cells in several important respects, such as the mutatome [51,52]. Finally, while there is a large number of human cell lines with various genotypes and phenotypes, there is only one murine cell line with different subclones [48]. To overcome this issue, human melanoma cells can be xenografted into so-called humanized mice, in which a functional human immune system is generated by introducing human CD34^+^ hematopoietic stem cells into mice previously subjected to gamma irradiation-induced myeloablation [45]. While this approach is relatively new and expensive, classic genetically defined predisposed mouse models provide useful information that complements *in vitro* and xenograft studies.

## 5. Genetically Engineered Mouse Models (GEM)

Mice rarely develop melanoma spontaneously, but can be genetically manipulated (transgenic and/or knockout mice) to do so by activating oncogenes relevant to human melanoma, such as mutant BRAF or mutant NRAS, and/or via inactivation of key tumor suppressors, including CDKN2A or PTEN, *i.e.*, they harbor defined genetic aberrations, which mimic the genetic lesions (or their consequences) that occur in human melanomas [45]. In these GEM tumor models, tumors normally occur spontaneously and—importantly—at their natural site. Unintended selection for cells that are resistant to apoptosis, an unavoidable problem with cell-line-based models, is obviated. Mice can also be manipulated to include other mutations to test their effects on drug sensitivity or resistance of the model melanomas. Unlike xenograft hosts, mice used for GEM models have a fully functional immune system, which impinges on tumor growth. They therefore serve as reliable and repeatable (every tumor has the same basal mutations) models to study the role of altered genes/pathways and the role of the immune system cells in melanoma biology and drug resistance [53,54]. The main challenge of utilizing GEM models is the cost and the effort involved.

It is also important to consider the different histology to human skin: Mouse melanocytes are located in the hair follicles and are mainly responsible for the pigmentation of the mouse fur. In contrast, in human skin there are interfollicular melanocytes, which are responsible for delivering pigment to the surrounding keratinocytes to protect epidermal cells from ultraviolet irradiation-induced genetic damage. Thus, melanomagenesis may not happen at the same site or may be different altogether in human and mice.

**Figure 6:**
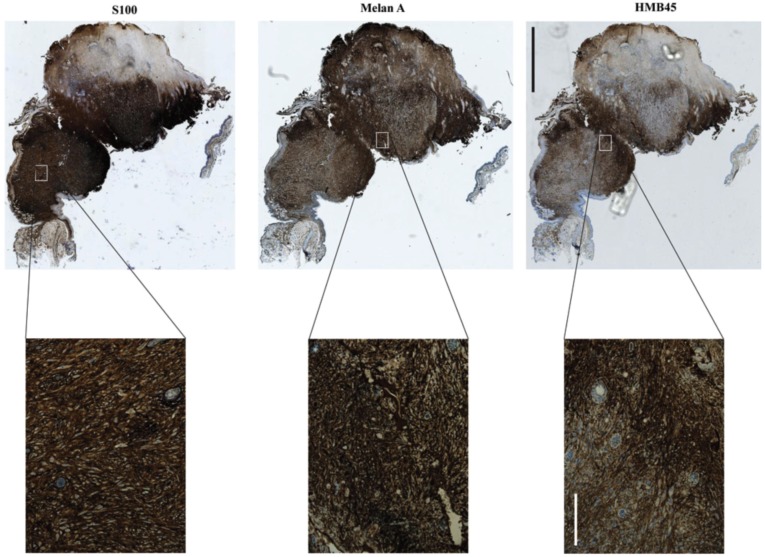
Spontaneous melanoma, which manifested on the ear of a UVB irradiated *Cdkn2a^−/−^*, Tyr-*HRAS* mouse. Staining positivity for all three markers (S100, Melan A and HMB45) as well as their pattern and localization are indicative of melanoma. Scale bars are 2 mm for the low (5×) and 200 μm for the high magnification (20×) images.

The first transgenic mouse melanoma model was the *Tyr-SV40* model. In this model *SV40*, which represses p53 and pRb, is under control of a tyrosinase promoter active throughout the melanocyte lineage and melanomas develop either spontaneously or after UV irradiation [55]. Lynda Chin and colleagues developed one of the first mouse melanoma models targeting more melanoma-specific genes: the *Cdkn2a^−/−^*, *Tyr-HRAS* model [56]. This model is null for both products of the *Cdkn2a* locus, p16^INK4A^ and p19^ARF^, mirroring this common lesion in human melanomas, and it carries the *HRAS* transgene under control of the tyrosinase promoter, activating the MAPK pathway, as occurs in human melanomas through *NRAS* or *BRAF* mutation. *Cdkn2a^−/−^*, *Tyr-HRAS* mice develop spontaneous melanoma (Figure 6) before other tumors in the majority of cases [56]. However, *Cdkn2a^−/−^*, *Tyr-HRAS* melanomas do not metastasize and other cancers, predominantly B cell lymphomas and soft-tissue sarcomas, also occur [57].

Dhomen and colleagues developed a melanoma model driven by *BRaf^V600E^*, however, the long latency required for the development of melanomas demonstrated that additional genetic lesions were required [58]. Most notably, Dankort and colleagues developed a mouse melanoma model in which *BRaf^V600E^* cooperates with *Pten* loss to induce metastatic melanoma [59]. This model provides a system to study features of melanoma metastasis and evaluate drugs, which could be used to prevent melanoma metastasis. The Wnt/β-catenin signaling pathway is frequently upregulated in melanoma but its functional implication is unclear. Damsky and colleagues studied the functional role of β-catenin by modulating it in the *BRaf^V600E^Pten^−/−^* model. They showed that β-catenin is the mediator of melanoma metastasis to lymph nodes and lungs. In addition to its role in metastasis, β-catenin levels control cell differentiation and regulate both the MAPK and the PI3K/AKT signaling pathways [60].

Locally invasive melanoma and distant metastatic phenotypes were reported in the *HGF/SF*-transgene *Ink4a^ARF−/−^*, *Tyr-N-Ras^Q61K^ INK4A^−/−^* and in the *BRaf ^V600E^ Pten^−/−^* mouse models of melanoma [59,61,62]. Inducible GEM model systems as well as the novel retrovirally-based gene transfer model (RCAS/TVA) permits temporal and spatial control of gene expression, which facilitates tumor progression and maintenance studies. Tumors evolve from mutations in developmentally normal cells in the context of an unaltered microenvironment, which closely mimics the context of human disease [45,63].

Chemical carcinogens, such as 7,12-dimethylbenz(a)anthracene (DMBA) and 12-O-tetradecanoylphobol-13-acetate (TPA), applied topically to the skin, have been used to induce melanoma in mice, or to decrease the latency of melanomagenesis. The application of chemical carcinogens can be replaced by UVB irradiation on the grounds that UVB exposure is part of the etiology of human melanoma and because of the ease to irradiate newborn mice evenly and consistently in contrast to the application of mutagens.

## 6. Other Animal Models

### 6.1. Fish Models

As outlined in the previous section, mouse models are commonly used in cancer research, since mice are physiologically most similar to humans, with directly comparable cell lineage and differentiation pathways. However, fish models of melanoma have several advantages over mouse models. Fish have a much shorter generation time, large number of progeny, low cost/small housing and there is the ability to do high throughput studies [64]. The first fish models of both spontaneous and induced melanoma formation were discovered in the genus *Xiphophorus* (platyfish and swordtails) [65,66,67]; Steven Kazianis was one of the pioneers in this field [68]. The spontaneous melanoma that developed in certain strains of *Xiphophorus* is histologically similar to human melanoma. These studies have shed light on several genes that are involved in melanoma development.

More recently, transgenic fish melanoma models have been developed in zebrafish *Danio rerio* [69] and in medaka *Oryzias latipes* [70]. Of these, especially, zebrafish have been gaining popularity recently as a model for melanoma as well as other cancers [71]. Due to the transparency of their embryos, which develop externally, high-resolution visualization of transplanted fluorescent melanoma cells *in vivo* is possible with relative ease [72]. Genetic manipulation in these fish is simple, with transgenes or morpholinos injected into the embryo (at the single cell or later developmental stages). The most famous use of zebrafish in melanoma was to demonstrate the role of the BRAF V600E mutation in nevus formation, and to show that an additional mutation was needed to develop melanoma [69]. Further, the zebrafish model has been proven to be useful for drug screening [64,73].

### 6.2. Avian and More Mammalian Models

In addition to the above-discussed mouse models, there is a variety of other mammalian melanoma models including Syrian hamster [74], swine [75,76], horse [77,78,79], and gray short-tailed opossum *Monodelphis domestica* [80].

Very elegant is the chick embryo melanoma model [81,82,83,84,85]. The neural tube transplant serves as a model for spontaneous neural crest migration; while there is spontaneous neural crest migration of melanoma cells, this is not the case for primary human melanocytes. The optic cup transplant serves as a model for melanoma invasion and the rhombencephalon transplant as a model for brain metastasis [85,86].

## 7. Conclusions

**Figure 7:**
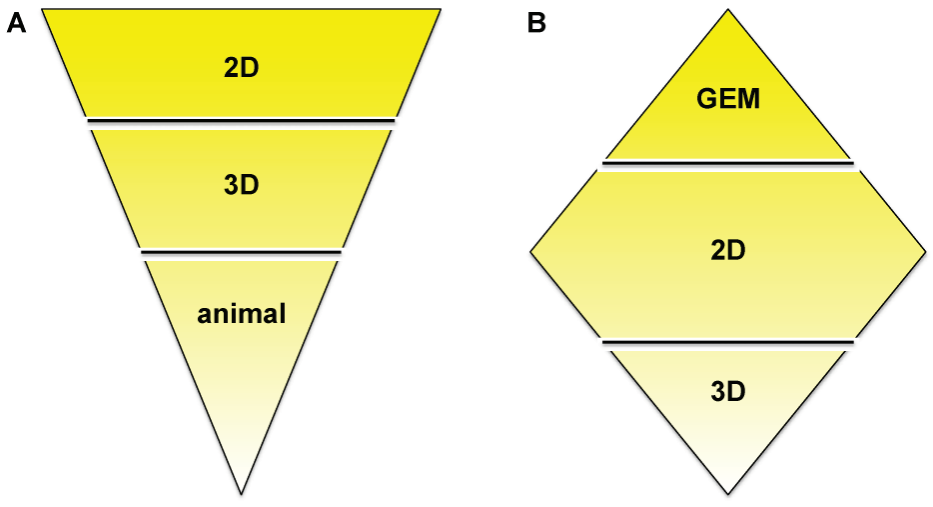
‘Upside-down triangle approach’ (**A**) and ‘diamond approach’ (**B**).

In order to accommodate all claims listed in the introduction—to recreate tumor progression, migration, invasion, its interaction with the microenvironment, drug delivery and bioavailability, but also ease of use and cost—we suggest a combination of multiple models as outlined in Figure 7. The ‘upside-down triangle approach’ (Figure 7A) is often used to perform drug discovery/development studies: Initial high-throughput screening in 2D culture using a high number of cell lines or primary cells and/or compounds or drug combinations. From the 2D experiments certain conditions of interest may be chosen for the next step, a semi-high-throughput screen in 3D culture. Based on data generated in 3D models, the final step should be the appropriate animal experiments. *In vitro* pre-screening should lead to the involvement of significantly fewer animals and therefore should provide a cost- and time-effective as well as ethically acceptable approach. An alternative is the ‘diamond approach’, in which cell lines can be isolated from a tumor growing in a GEM and characterized in 2D and then 3D culture. Similarly, either approach can be applied for mechanistic studies. The decision on which approach to apply may be guided by the pros and cons discussed in this review.
